# Own-race and own-age biases facilitate visual awareness of faces under interocular suppression

**DOI:** 10.3389/fnhum.2014.00582

**Published:** 2014-08-01

**Authors:** Timo Stein, Albert End, Philipp Sterzer

**Affiliations:** ^1^Center for Mind/Brain Sciences, CIMeC, University of TrentoRovereto, Italy; ^2^Department of Psychiatry, Charité Universitätsmedizin BerlinBerlin, Germany; ^3^Berlin School of Mind and Brain, Humboldt-Universität zu BerlinBerlin, Germany; ^4^DFG Research Unit Person Perception, Friedrich Schiller University of JenaJena, Germany; ^5^Department of Systems Neuroscience, University Medical Center Hamburg-EppendorfHamburg, Germany; ^6^Bernstein Center for Computational NeuroscienceBerlin, Germany

**Keywords:** face perception, face detection, visual awareness, race, age, interocular suppression, continuous flash suppression

## Abstract

The detection of a face in a visual scene is the first stage in the face processing hierarchy. Although all subsequent, more elaborate face processing depends on the initial detection of a face, surprisingly little is known about the perceptual mechanisms underlying face detection. Recent evidence suggests that relatively hard-wired face detection mechanisms are broadly tuned to all face-like visual patterns as long as they respect the typical spatial configuration of the eyes above the mouth. Here, we qualify this notion by showing that face detection mechanisms are also sensitive to face shape and facial surface reflectance properties. We used continuous flash suppression (CFS) to render faces invisible at the beginning of a trial and measured the time upright and inverted faces needed to break into awareness. Young Caucasian adult observers were presented with faces from their own race or from another race (race experiment) and with faces from their own age group or from another age group (age experiment). Faces matching the observers’ own race and age group were detected more quickly. Moreover, the advantage of upright over inverted faces in overcoming CFS, i.e., the face inversion effect (FIE), was larger for own-race and own-age faces. These results demonstrate that differences in face shape and surface reflectance influence access to awareness and configural face processing at the initial detection stage. Although we did not collect data from observers of another race or age group, these findings are a first indication that face detection mechanisms are shaped by visual experience with faces from one’s own social group. Such experience-based fine-tuning of face detection mechanisms may equip in-group faces with a competitive advantage for access to conscious awareness.

## Introduction

Faces are a rich source of important social information. Before this information can be accessed, however, the presence of a face in a visual scene needs to be detected. While much research has examined how we identify and remember individual faces, surprisingly little is known about the perceptual mechanisms underlying the initial detection of a face. Most classical theories of face perception only deal with the perceptual and cognitive operations that are carried out after a face has been detected in a scene (Bruce and Young, 1986; Burton et al., 1999; Haxby et al., 2000). It appears plausible, however, that face detection is supported by perceptual mechanisms distinct from those analyzing specific facial properties such as identity, because face detection and face recognition have fundamentally different computational goals (Tsao and Livingstone, 2008): Whereas recognition mechanisms need to extract facial information that distinguishes individual faces, detection mechanisms need to be sensitive to information that is common to all faces. Indeed, there is evidence for a dissociation between face detection and face recognition in prosopagnosic individuals who show severe deficits in face discrimination but perform well in face detection tasks (de Gelder and Rouw, 2000; Le Grand et al., 2006; Garrido et al., 2008). Accordingly, recent models of face perception have incorporated a distinct initial stage of face detection in a hierarchy of face processing stages (de Gelder et al., 2003; Johnson, 2005; Duchaine and Nakayama, 2006; Tsao and Livingstone, 2008).

How could face detection mechanisms localize regions in a visual scene that contain a face? Because all faces share the same global structure, face detection can efficiently be achieved by matching the visual input to an internal representation corresponding to the structure of a prototypical face (Lewis and Ellis, 2003). Although the exact nature of this face representation or face template is currently unknown, it appears likely that face detection mechanisms are tuned to the spatial configuration of facial parts that are invariant across different face exemplars (e.g., two eyes above nose above mouth; McKone et al., 2007; Tsao and Livingstone, 2008). When these “first-order relations” are distorted by turning faces upside down, face detection performance declines significantly (Purcell and Stewart, 1988; Lewis and Edmonds, 2003; Garrido et al., 2008). Because upright and inverted faces are physically identical, this face inversion effect (FIE) supports the notion that face detection mechanisms rely on information about the common spatial configuration of facial parts.

**Figure 1:**
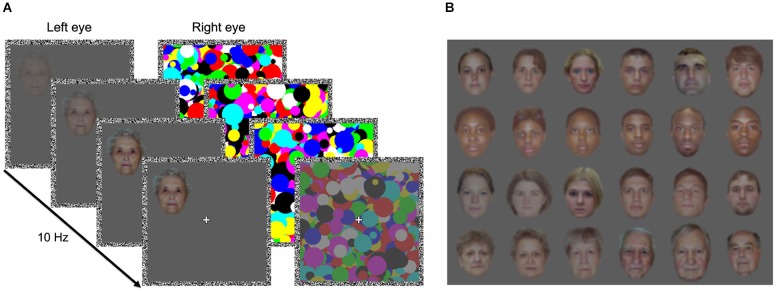
**Breaking continuous flash suppression (b-CFS) paradigm and face stimuli. (A)** Schematic of an example b-CFS trial. An upright or an inverted face was gradually introduced to one eye. To render the face target invisible for the first seconds of each trial through interocular suppression, CFS masks flashing at 10 Hz were presented to the other eye. The contrast of the CFS masks was slowly ramped down over the course of each trial. Participants indicated as quickly and accurately as possible on which side of fixation the target or any part of the target became visible. **(B)** Example face stimuli. Rows from top to bottom: young Caucasian adults from the race experiment, young Black adults from the race experiment, young Caucasian adults from the age experiment, and old Caucasian adults from the age experiment.

A particularly striking demonstration of the impact of face inversion on detection performance comes from experiments using strong interocular suppression induced by continuous flash suppression (CFS; Tsuchiya and Koch, 2005). In CFS, a train of high-contrast, contour-rich masks flashed into one eye can render a face photograph projected to the other eye invisible for up to several seconds (see Figure 1A). The time faces need to overcome suppression and gain access to awareness is strongly modulated by their orientation: Upright faces break into awareness much more quickly than inverted faces (Jiang et al., 2007; Yang et al., 2007; Stein et al., 2011a). This FIE in breaking continuous flash suppression (b-CFS) is larger than the effect of inversion on b-CFS for most other objects (Stein et al., 2012b), indicating that the FIE reflects face-specific detection mechanisms (Zhou et al., 2010a). Thus, comparing the duration of perceptual suppression of physically identical upright and inverted faces under CFS represents a powerful and well-controlled method for studying mechanisms of face detection.

With this approach, we have recently found evidence that face detection mechanisms are broadly tuned to register all visual information that could be indicative of a face: Even simple schematic head-shaped patterns consisting of three dark blobs were detected more quickly when the spatial arrangement of these blobs resembled the face-like configuration of two eyes above the mouth than when this configuration was inverted (Stein et al., 2011b). Interestingly, these face-like patterns also preferentially attract the gaze of newborns in their first few days of life (e.g., Farroni et al., 2005). Thus, it is possible that relatively hard-wired face detection mechanisms respond to all visual patterns that contain face-like first-order relations among face-like parts (also see Tomalski et al., 2009a,b). However, there is also evidence that face detection mechanisms can be modified by visual experience and respond optimally to those faces that have been encountered most frequently. First, the inversion effect for schematic face-like patterns is smaller than for naturalistic face photographs (Stein et al., 2011b). Second, Gobbini et al. (2013) recently reported that upright faces of close friends overcame CFS more quickly than upright faces of strangers. However, as this b-CFS study did not include inverted faces, faster detection of highly familiar faces could have been due to uncontrolled differences in low-level physical stimulus characteristics.

To better understand the tuning properties of face detection mechanisms, in the present study we used b-CFS to measure detection performance and inversion effects for faces from the observer’s own race or from another race (race experiment) and for faces from the observer’s own age group or from another age group (age experiment). While it is well established that the greater experience we have with people from our own race and age group is associated with better recognition memory for own-race and own-age faces (Meissner and Brigham, 2001; Rhodes and Anastasi, 2012), it is unknown whether own-race and own-age biases facilitate the initial detection of a face. Faces from different races and age groups have identical first-order relations among facial parts, but differ in face shape and surface reflectance properties (Berry and McArthur, 1986; Hill et al., 1995). Thus, if face detection mechanisms were relatively hard-wired and broadly tuned to fit all visual patterns having face-like first-order relations (Stein et al., 2011b), the FIE should be of similar size for all face categories. Alternatively, if the mechanisms supporting visual awareness were shaped by experience and thus optimally tuned to more frequently encountered faces (Gobbini et al., 2013), the FIE in b-CFS should be larger for same-race and same-age faces than for other-race and other-age faces.

## Method

### Participants

Fourteen Caucasian students (12 female, age range 20–35 years, *M* = 24.9 years, *SD* = 4.5 years) participated for course credit or monetary compensation. All participants had normal or corrected-to-normal vision and were naïve to the purpose of the study. The study protocol was approved by the Charité ethics committee.

### Display and stimuli

Participants viewed a CRT screen from a distance of 50 cm through a mirror stereoscope, such that each eye was presented with one of two fusion contours (11.0° × 11.0° of visual angle) consisting of white noise pixels (width 0.5°). Because the precise luminance was not critical to our research question, i.e., the comparison of physically identical upright and inverted faces, we did not linearize the monitor output. Faces were presented on a mid-gray background within these fusion contours, with the remainder of the screen being black. In the center of each fusion contour a fixation cross (0.7° × 0.7°) was displayed and participants were asked to maintain stable fixation throughout each experimental block. We created multicolored Mondrian-like CFS masks (10.0° × 10.0°) consisting of randomly arranged circles (diameter 0.4°–1.8°) and selected 120 colored face photographs from the “Center for Vital Longevity Face Database” (Minear and Park, 2004).

In the *race experiment*, we used 30 photographs of young Caucasian adults (15 female, age range 18–27 years, *M* = 21.9 years, *SD* = 2.6 years) and 30 photographs of young Black adults (15 female, age range 18–30 years, *M* = 22.8 years, *SD* = 3.4 years). In the *age experiment*, we used another set of 30 photographs of young Caucasian adults (age range 18–27 years, *M* = 22.0 years, *SD* = 2.1 years) and 30 photographs of older Caucasian adults (age range 65–91 years, *M* = 74.9 years, *SD* = 7.1 years). All non-facial features were cropped and the images were resized to approximately 3.5° × 4.0°, retaining some variability in face size (Figure 1B). Then the stimuli’s luminance and RMS contrast were adjusted (based only on the monitor’s input values, as we did not linearize the monitor output), separately for each RGB channel (in the race experiment, the RMS contrast was slightly higher than in the age experiment). To preserve each face’s original color composition, we computed the relative contribution of each RGB channel to the luminance of the original stimulus, which served as a weighting factor for each RGB channel. These weighting factors were then used to normalize each RGB channel’s luminance proportionally to its weight in the original image.

Note that a precise matching of low-level stimulus characteristics was not critical to our research question, as we compared breakthrough from CFS for physically identical stimuli shown in upright and inverted orientations. Even for grayscale face stimuli, it is virtually impossible to equate all low-level physical stimulus properties that may influence b-CFS (e.g., Yang et al., 2007; Stein and Sterzer, 2012; Stein et al., 2012b). For colored face photographs, this problem is further complicated by the non-trivial interaction of color channels. Therefore, we did not attempt to precisely match the color photographs used in the present experiments, but only sought to achieve roughly similar overall suppression durations.

### Procedure

Participants performed a standard b-CFS localization task: After a 1-s fixation period, CFS masks flashing at 10 Hz were presented to one randomly selected eye, while a face was gradually introduced to the other eye by ramping up its contrast over the first second of each trial. Beginning 2.1 s after trial onset, the contrast of the CFS masks was linearly ramped down to zero over 6.9 s. The face was presented until response or for a maximum trial length of 10 s. On each trial, a face was centered at a random vertical position (maximally 2.6° below or above the fixation cross) in the left or the right half of the fusion contour (2.9° from the fixation cross). Participants were informed about the presentation of upright and inverted face targets and were asked to press the left or the right arrow key on the keyboard to indicate as fast and accurately as possible on which side of fixation a face or any part of a face emerged from suppression.

Both the race and the age experiment consisted of 240 trials (separated by mandatory breaks after 80 and 160 trials). We counterbalanced the order of the two experiments across participants. In both experiments each combination of two face categories (race experiment: Caucasian faces, Black faces; age experiment: young faces, old faces), two face orientations (upright and inverted), two eyes for face presentation, and 30 face exemplars was presented once. The order of trials was randomized.

### Analysis

We excluded trials with incorrect responses from the analysis (race experiment: 1.7% of all trials, age experiment: 2.1% of all trials). As an effect size estimate for the paired *t*-tests we report Cohen’s *d* as the pooled mean divided by the standard deviation.

## Results

### Race experiment

**Figure 2:**
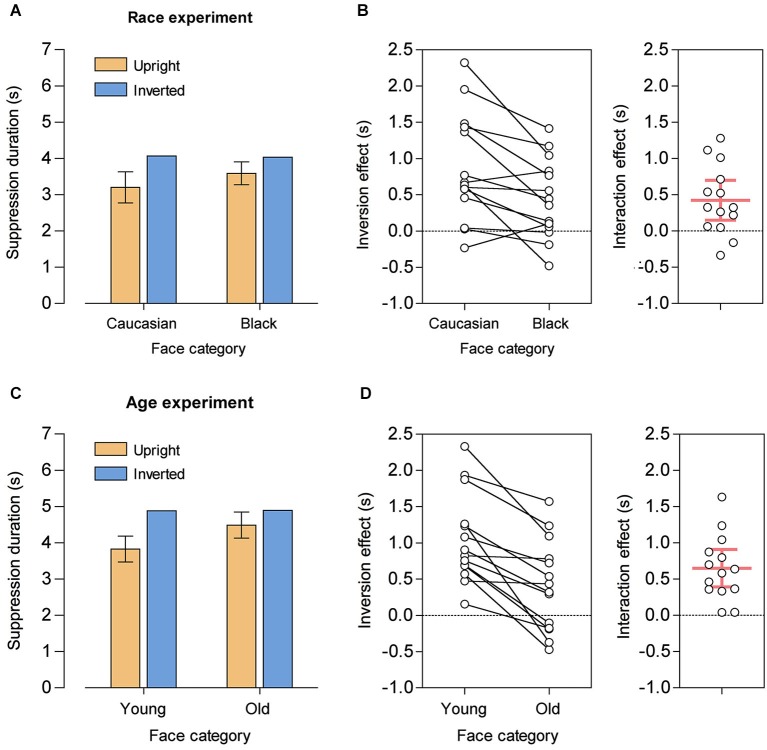
**Results from the race experiment (A, B) and from the age experiment (C, D)**. **(A)** Mean suppression durations for upright and inverted Caucasian and Black faces. Error bars show 95% CIs for the mean difference between upright and inverted faces (that is, 95% CIs of the face inversion effects), separately for Caucasian and Black faces. **(B)** Individual subject data. Left panel: Inversion effects (difference in mean suppression durations between upright and inverted faces) for Caucasian and Black faces. Right panel: Interaction effect, with positive values reflecting a larger inversion effect for Caucasian faces than for Black faces. The red horizontal bar denotes the group mean and the red vertical error bar represents the 95% CI. **(C)** Mean suppression durations for upright and inverted young and old faces. Error bars show 95% CIs for the mean difference between upright and inverted faces, separately for young and old faces. **(D)** Individual subject data. Left panel: Inversion effects for young and old faces. Right panel: Interaction effect, with positive values reflecting a larger inversion effect for young faces than for old faces. The red horizontal bar denotes the group mean and the red vertical error bar represents the 95% CI.

A repeated-measures ANOVA with the factors race (Caucasian, Black) and orientation (upright, inverted) on the mean suppression durations yielded a significant main effect of orientation, *F*_(1, 13)_ = 15.98, *p* = 0.002, $\eta_{p}^{2}$ = 0.55, reflecting overall shorter suppression durations for upright faces, and a significant race-by-orientation interaction, *F*_(1, 13)_ = 11.05, *p* = 0.005, $\eta_{p}^{2}$ = 0.46. The main effect of race did not reach statistical significance, *F*_(1, 13)_ = 3.67, *p* = 0.078, $\eta_{p}^{2}$ = 0.22. Compared to their inverted counterparts, suppression durations were shorter for both upright Caucasian faces, *t*_(13)_ = −4.33, *p* = 0.001, *d* = 1.16 (*M* = −865 ms, *SD* = 747 ms, 95% CI [−1296 ms, −433 ms]), and upright Black faces, *t*_(13)_ = −3.01, *p* = 0.010, *d* = 0.80 (*M* = −442 ms, *SD* = 550 ms, 95% CI [−760 ms, −124 ms]). Importantly, however, the significant interaction demonstrated that the FIE was significantly larger for Caucasian faces, i.e., for own-race faces (*M* = 423 ms, *SD* = 476 ms, 95% CI [148 ms, 697 ms], see Figures 2A,B).

### Age experiment

For the age experiment, a repeated-measures ANOVA with the factors age (young, old) and orientation revealed a similar pattern of results. There was a significant main effect of age, *F*_(1, 13)_ = 26.08, *p* < 0.001, $\eta_{p}^{2}$ = 0.67, reflecting overall shorter suppression durations for young faces, a significant main effect of orientation, *F*_(1, 13)_ = 22.09, *p* < 0.001, $\eta_{p}^{2}$ = 0.63, and a significant age-by-orientation interaction, *F*_(1, 13)_ = 29.65, *p* < 0.001, $\eta_{p}^{2}$ = 0.70. Again, compared to their inverted counterparts, suppression durations were shorter for both upright young faces, *t*_(13)_ = −6.39, *p* < 0.001, *d* = 1.71 (*M* = −1055 ms, *SD* = 618 ms, 95% CI [−1413 ms, −699 ms]), and for upright old faces, *t*_(13)_ = −2.42, *p* = 0.031, *d* = 0.65 (*M* = −406 ms, *SD* = 628 ms, 95% CI [−768 ms, −43 ms]). Crucially, the significant interaction demonstrated a larger FIE for young faces, i.e., for own-age faces (*M* = 650 ms, *SD* = 446 ms, 95% CI [392 ms, 907 ms], see Figures 2C,D).

### Linear mixed effects analyses

To account for variability in suppression durations between face exemplars, we also performed linear mixed effects analyses using the lme4 package (Bates et al., 2012) for R (R Core Team) on the raw suppression durations and, due to their positive skew, also on log-transformed suppression durations. These analyses had random intercepts for participants and for individual face exemplars. Reduced models containing only these random effects of participants and face exemplars were tested against models including fixed effects of orientation (upright, inverted) or face category (race experiment: Caucasian, Black; age experiment: young, old) using likelihood ratio tests. To test for the interaction effect, models with the orientation-by-category interaction were compared to models with the two fixed factors only.

For the analyses of raw suppression durations from the race experiment, the comparison of the reduced model with the model containing the additional fixed factor of orientation was significant, *χ*^2^(1) = 102.82, *p* < 0.001, while the comparison with the model containing the additional fixed factor of face category did not reach significance, *χ*^2^(1) = 1.31, *p* = 0.252. Most importantly, the interaction was significant, *χ*^2^(1) = 10.76, *p* = 0.001. The results of the analyses of log-transformed suppression durations from the race experiment were similar, for orientation, *χ*^2^(1) = 98.86, *p* < 0.001, for face category, *χ*^2^(1) = 2.15, *p* = 0.143, and for the interaction, *χ*^2^(1) = 10.20, *p* = 0.001.

For the age experiment, analogous analyses of raw suppression durations revealed a significant effect of orientation, *χ*^2^(1) = 94.94, *p* < 0.001, a significant effect of face category, *χ*^2^(1) = 4.18, *p* = 0.041, and a significant interaction effect, *χ*^2^(1) = 19.17, *p* < 0.001. Finally, a similar pattern of results was obtained for the analyses of log-transformed suppression durations from the age experiment, for orientation, *χ*^2^(1) = 93.914, *p* < 0.001, for face category, *χ*^2^(1) = 5.14, *p* = 0.023, and for the interaction, *χ*^2^(1) = 18.24, *p* < 0.001. Thus, the results from the linear mixed effects analyses were consistent with the outcome of the standard repeated-measures ANOVA reported above, meaning that the effects persisted after accounting for variability across individual face exemplars.

### Similarity of suppression durations for inverted faces

Additional *post hoc* *t*-tests showed that for inverted faces suppression durations did neither differ between own-race and other-race faces nor between own-age and other-age faces, both *t* < 1. The similarity of suppression durations for inverted faces can be regarded as an a posteriori validation of our attempt to match faces in terms of low-level physical stimulus characteristics. By contrast, when displayed in upright orientation, suppression durations were shorter for own-race faces compared to other-race faces, *t*_(13)_ = 3.57, *p* = 0.003, *d* = 0.95 (*M* = −389 ms, *SD* = 407 ms, 95% CI [−624 ms, −153 ms]), as well as for own-age faces compared to other-age faces, *t*_(13)_ = −6.34, *p* < 0.001, *d* = 1.69 (*M* = −660 ms, *SD* = 389 ms, 95% CI [−884 ms, −435 ms]). Thus, the increased FIE for own-race and own-age faces most likely reflected a greater advantage of upright over inverted faces in gaining access to awareness.

### Experimental order and own-race vs. own-age bias

Because we used a within-subjects design, it is possible that the temporal order of the experiments affected our results. In particular, young Caucasian faces were included in both experiments. Thus, after the first experiment observers might have been accustomed to the presentation of the specific face categories used in the first experiment, of which only young Caucasian faces were repeated in the second experiment (albeit using different exemplars). We therefore conducted an additional mixed ANOVA with the between-subjects factor experimental order (race experiment first, age experiment first) and the within-subjects factors experiment (race, age), face category (own, other), and orientation. There was no significant four-way interaction and there were no significant three-way interactions with experimental order (all *F* < 1), indicating that the difference in FIEs for own- and other faces was similar for the first and the second experiment, both for the race experiment (first, *M* = 423 ms, *SD* = 477 ms; second, *M* = 423 ms, *SD* = 513 ms), as well as for the age experiment (first, *M* = 528 ms, *SD* = 427 ms; second, *M* = 771 ms, *SD* = 464 ms). Furthermore, because the three-way interaction between experiment, face category, and orientation was not significant, *F*_(1, 12)_ = 2.76, *p* = 0.123, $\eta_{p}^{2}$ = 0.19, there was no evidence for differences in the strength of the FIE modulation by the own-race and the own-age bias.

## Discussion

Upright faces have a robust advantage over inverted faces in overcoming CFS and breaking into awareness (e.g., Jiang et al., 2007; Yang et al., 2007; Stein et al., 2011a,b). This FIE demonstrates the sensitivity of detection mechanisms to the global facial structure, i.e., the spatial configuration or first-order relations of face parts, which is disrupted in inverted faces (Purcell and Stewart, 1988; Lewis and Edmonds, 2003; Lewis and Ellis, 2003). Most likely, the FIE reflects face-specific detection mechanisms, as the impact of inversion on b-CFS is greater for faces than for most other object categories (Zhou et al., 2010a; Stein et al., 2012b). The present findings show that face detection mechanisms are not only sensitive to face orientation, but also to comparably subtle differences in face shape and surface reflectance. Young Caucasian adults detected faces of their own race and age group more quickly than young Black faces and old Caucasian faces. This advantage of upright own-race and own-age faces over upright other-race and other-age faces is unlikely to merely reflect differences in low-level physical stimulus properties (e.g., higher contrast at the hairline in young Caucasian faces), because we did not obtain similar differences in suppression durations when the same faces were inverted. Moreover, the advantage of upright over inverted faces in gaining access to awareness, i.e., the FIE, was increased for own-race and own-age faces. This indicates that configural face processing at the initial detection stage can be influenced by facial properties that differ between faces from different race and age groups, namely by differences in face shape and surface reflectance (including, e.g., albedo, hue, texture; Russell et al., 2007).

This influence of face shape and facial surface reflectance properties on the FIE in simple detection has implications for our understanding of the perceptual mechanisms involved in visual awareness of faces. It has been proposed that faces are detected by matching the visual input to a deformable internal representation of a prototypical face (Lewis and Ellis, 2003). A poor match between this (upright) face template and inverted faces could account for the FIE. We have recently provided evidence that this face template only represents the prototypical first-order and ordinal luminance contrast relationships among facial parts that are shared by all faces under natural lighting conditions (Stein et al., 2011b). This account cannot explain the increased FIE for own-race and own-age faces. Rather, the present findings indicate that the face template guiding detection holds a more detailed representation of a prototypical face, containing information about face shape and surface reflectance properties.

### Limitations of the present one-group design for interpreting own-race and own-age biases

It seems natural to interpret these own-race and own-age biases as indicative that experience with people from one’s own race and age group finely tunes detection mechanisms to faces from one’s own social categories. However, to be precise, our data only show that for young Caucasian observers the advantage of upright over inverted faces in gaining access to awareness is larger for young Caucasian faces than for young Black and old Caucasian faces. While the comparison of upright and inverted faces rules out that low-level stimulus differences caused this pattern of results, our findings do not yet establish unequivocal evidence for a fine-tuning of face detection mechanisms to one’s own social categories. For this, it would have been necessary to show a reversed pattern of results with young Black or old Caucasian observers. As we could not collect data from these groups of observers due to logistic challenges, testing for this crossover interaction remains an important avenue for future studies. Thus, our findings leave open the possibility that face detection mechanisms are generally tuned to detect young faces with light skin color (e.g., Rhodes, 2006), independent of the observer’s own social group membership and visual experience.

Although we cannot exclude this possibility, in the light of other recent evidence for the influence of experience on face detection (Gobbini et al., 2013) we consider an experience-based mechanism a more likely explanation for the present findings. This interpretation would dovetail with recent accounts of own-race and own-age effects in face recognition memory. For example, the “experience-based holistic account” by Rossion and Michel (2011) holds that memory deficits for other-race (and potentially other-age) faces result from a poor match between the faces’ unfamiliar morphology and an experience-derived template representing the global structure of an average face. Consequently, information diagnostic for discriminating individual out-group faces is processed in a less holistic, more piecemeal fashion, and thus less efficiently (Tanaka et al., 2004; Michel et al., 2006; de Heering and Rossion, 2008). In support of this notion, the detrimental effect of inversion on recognition memory is reduced for other-race (Rhodes et al., 1989; Hancock and Rhodes, 2008; Rhodes et al., 2009) and other-age (Kuefner et al., 2008) faces. Adopting this view, face detection could involve fitting the visual input to a face template that is shaped by the observer’s specific experience with faces. The goodness of fit between the visual input and this experience-based face template would determine detection performance and equip faces from one’s own social categories with an advantage in gaining access to awareness.

### Unconscious processing of facial race and age or mere tuning of face detection mechanisms?

In the present study we recorded the duration of perceptual suppression as a marker of different perceptual sensitivities to faces from the observer’s own and other race or age group. A number of previous b-CFS studies went one step further and took a difference in breakthrough from CFS as evidence for differential unconscious processing occurring while stimuli are still suppressed (e.g., Jiang et al., 2007; Stein et al., 2011c). Most commonly, this inference rested on the comparison to a binocular control condition not involving CFS. However, we have recently provided theoretical and empirical reasons that question the logic of relying on a control condition to infer unconscious processing under interocular suppression (Stein et al., 2011a; Stein and Sterzer, 2014). Therefore, following other recent b-CFS studies (e.g., Yang et al., 2007; Tsuchiya et al., 2009; Zhou et al., 2010b; Stein et al., 2014), here we did not include a binocular control condition and do not claim that differences in suppression durations necessarily reflect differential unconscious processing of facial race and age under CFS.

One may still argue that, because faces presented under CFS went undetected for several seconds, differences in breakthrough need to reflect unconscious processing of facial race and age during this long period of subjective invisibility. However, comparable detection latencies can be obtained with techniques other than CFS, such as difficult visual search for faces (e.g., Garrido et al., 2008). Thus, the mere length of overall response times cannot be taken as proof of unconscious processing. To provide unequivocal evidence for unconscious processing, one would need to demonstrate that a subliminal stimulus that is rendered permanently invisible still has some influence on a measure of perceptual or cognitive processing (Stein et al., 2011a; Stein and Sterzer, 2014). Adopting this dissociation logic, neuroimaging studies revealed that neural responses differentiate between invisible faces and non-face stimuli (e.g., Jiang and He, 2006; Sterzer et al., 2008, 2009; for a review see Sterzer et al., 2014). There is only limited evidence, however, for specific facial features being processed unconsciously (Adams et al., 2010; Xu et al., 2011). Most studies indicate that the representation of facial shape, gender, identity, expression, and eye gaze requires awareness (Moradi et al., 2005; Shin et al., 2009; Yang et al., 2010; Amihai et al., 2011; Stein and Sterzer, 2011; Stein et al., 2012a). Amihai et al. (2011) found that faces rendered invisible through CFS failed to induce race adaptation aftereffects, indicating that there is no unconscious processing of facial properties that discriminate faces from different races. It thus appears more likely that the own-race and own-age biases observed in the present study reflect processing differences at the transition to awareness (cf. Gayet et al., 2014), that is, differences in stimulus detectability (Stein et al., 2011a; Stein and Sterzer, 2014).

### Possible neural mechanisms

Previously, we found evidence for face detection mechanisms in adult observers being broadly tuned to all head-shaped visual patterns with two dark blobs over one dark blob on a lighter background (Stein et al., 2011b), similar to the face-like stimuli that optimally drive newborns’ orienting behavior (e.g., Farroni et al., 2005). This finding led us to speculate that the initial detection of a face might rely on an inborn subcortical face detection pathway involving the superior colliculus, pulvinar, and the amygdala (de Gelder et al., 2003; Johnson, 2005; Nguyen et al., 2013). The present findings appear inconsistent with such a coarsely tuned subcortical face detection pathway, because the processing of relatively subtle differences in face shape and surface reflectance likely requires more elaborate cortical visual processing. Indeed, face-sensitive cortical visual regions such as fusiform and occipital face areas exhibit differential responses to own- and other-race faces (Golby et al., 2001; Feng et al., 2011; Natu et al., 2011) and, possibly, to own- and other-age faces (Ebner et al., 2013). Moreover, the impact of face inversion on the early face-sensitive event-related potentials N170 is larger for own- than other-race faces (Vizioli et al., 2010). Consistent with the present findings, this suggests that early cortical markers of structural face encoding are finely tuned to own-race faces. One important task for future studies is to directly relate these neural measures of face processing to facilitated awareness of own-race and own-age faces. Another interesting direction for future neuroimaging work is to determine whether cortical responses to faces suppressed through CFS (Jiang and He, 2006; Sterzer et al., 2008, 2009) distinguish faces from different race and age groups without awareness.

### Conclusion

In conclusion, the modulation of FIEs by race and age revealed in the present study demonstrates that the perceptual mechanisms governing awareness of faces are not only sensitive to the spatial configuration of facial parts, but also to variations in face shape and surface reflectance properties. These findings show that face detection mechanisms are more complex than previously thought and provide a first indication that experience fine-tunes the earliest levels of visual processing to faces from our own social groups.

## Conflict of interest statement

The authors declare that the research was conducted in the absence of any commercial or financial relationships that could be construed as a potential conflict of interest.
